# Association between transferred embryos and multiple pregnancy/live birth rate in frozen embryo transfer cycles: A retrospective study

**DOI:** 10.3389/fendo.2022.1073164

**Published:** 2023-01-05

**Authors:** Xian Wu, Wen-jie Zhou, Bu-fang Xu, Qian Chen, Lan Xia, Shen Zhao, Hui-hui Xu, Ai-jun Zhang, Zhi-hong Niu

**Affiliations:** Department of Obstetrics and Gynecology, Ruijin Hospital, Shanghai Jiao Tong University School of Medicine, Shanghai, China

**Keywords:** assisted reproductive technology, blastocyst, embryo transfer, live birth rate, pregnancy outcomes, retrospective cohort study

## Abstract

**Background:**

Physicians need an appropriate embryo transfer strategy to address the challenge of reducing multiple birth rates, while maintaining the couples’ live birth rate during assisted reproductive technology.

**Methods:**

We included 10,060 frozen embryo transfer cycles from January 2015 to March 2020 in reproductive medical center of Ruijin hospital, Shanghai, China. Patients were grouped according to the number and grade of cleavage-stage embryo or blastocysts transferred. Live birth rate and multiple live birth rate were compared among groups of women of different ages. Multivariable logistic regression models were used to estimate the risk of multiple live birth using different combinations of transferred embryos.

**Results:**

The transfer of double good-quality embryos was an independent predictor for multiple birth in women aged <30 years and those aged 36−39 years [<30 years: aOR =1.54 (95% CI: 1.14−2.06, P < 0.01); 36−39 years: aOR =1.84 (95% CI: 1.0−3.4, P < 0.01)]. Further, for women aged <36 years, the transfer of good-quality + poor-quality blastocysts was an independent predictor for multiple birth rate [<30 years: aOR=2.46 (95% CI: 1.45−4.18, P < 0.01); 31−35 years: aOR =4.45 (95% CI: 1.97−10.06, P < 0.01)].

**Conclusions:**

Single-good-quality blastocyst transfer is recommended for women of all ages. When good-quality cleavage embryos are available, the choice of single or double embryo transfer with good- or average-quality embryo should depend on the age of women. Double embryo transfer with the highest possible grade of embryos is recommended for women aged ≥40 years.

## Introduction

With improvements in assisted reproductive technology (ART), the strategy of single embryo transfer (SET) has been promoted in many countries, since the goal of ART is to produce a healthy baby. According to a study, SET can greatly decrease multiple pregnancy (MP) rates from 26−29% to 2%, and results in lower risks of miscarriage, preterm delivery, morbidity, and mortality of mothers and children (1). Many studies have shown that the risk of live birth is lower in the SET cycle than in the DET cycle (2–4). According to systematic reviews which included five randomized trials on pregnancy outcomes of SET and DET in fresh IVF cycle, the live birth rate (LBR) per cycle with DET was significantly higher than that with SET (OR: 2.10, 95% CI: 1.65 to 2.66), with MP rate being significantly lower in women who had SET compared with DET (OR: 0.04, 95% CI: 0.01- 0.11) (5). However, there are also data suggesting that a more liberal use of SET does not lead to a fall in LBR per cycle (6, 7). It was suggested that SET strategy has minimum impact on overall outcomes when it is applied in women with a better prognosis (8, 9), such as those younger than 35 years old, or having good quality blastocyst to be transferred. Thus, embryo transfer strategy should be personalized which was associated not only with LBR, but also with cumulative live birth rate (CLBR).

The comparison of LBR in SET and DET in frozen embryo transfer (FET) cycles has been reported by others (7). In this study, we conducted a larger retrospective analysis to investigate both LBR and multiple birth rate (MBR) under the combination of several parameters (DET/SET + embryo quality at different stages + women’s age), providing more accurate and valuable information to develop better cost-effective embryo transfer strategies.

## Materials and methods

### Ethics statement

The study protocol was approved by the Ethics Committee (Institutional Review Board) of Shanghai Ruijin Hospital.

### Participants

In this retrospective cohort study, we included 10,600 FET cycles (7631patients) performed between January 2015 and March 2020 at the reproductive medical center of Ruijin Hospital affiliated with Shanghai Jiao Tong University School of Medicine. Details of ovulation induction, embryo culture and evaluation, and embryo freeze-thaw have been described in our previous articles (10, 11).

### Endometrial preparation

Artificial hormone replacement therapy (HRT) cycles were used to prepare the endometrium. Briefly, estradiol valerate at a dose of 4–6 mg/day was started from day 2 of the menstrual cycle, and endometrial thickness was examined by ultrasound scan on days 12–14. If the thickness was ≥7 mm, progesterone gel (90 mg/day; Crinone, MERCK) was administered. Cleavage embryos were transferred on day 4, and blastocyst transfer was performed on the sixth day of progesterone supplementation. The administration of estradiol valerate and progesterone was continued until 10 weeks of gestation if pregnant.

### Patient groups

The patients were grouped according to the number and grade of embryos transferred, as detailed in **Table 1**. Embryo grading was performed just before embryo transfer using an Olympus microscope with 200× magnification. The score of cleavage embryos was based on the following three criteria (12): (A) Blastomere number (BL): 4 BL=1, 5 BL=2, 6–7 BL=3, and 8–10 BL=4; (B) Fragmentation (FR): <5%=4, 5–10%=3, 11–25%=2, 26–50%=1, and > 50%=0; and (C) symmetry (SY): perfect symmetry=score 1, and asymmetry=score 0. The score for an embryo was the sum of BL, FR, and SY. The group criteria were as follows: poor-quality embryo (PQE)= score 5; average-quality embryo (AQE)= score 6–7; and good-quality embryo (GQE)= score ≥8.

**Table 1:** Patients groups based on the number and grade of embryos transferred.

Cleavage-stage	DET (double embryo transfer)				SET (single embryo transfer)
	**GQE**	**AQE**		**PQE**	**/**
**GQE**	**B1**	**/**		**/**	**A1**
**AQE**	**B2**	**C1**		**/**	**A2**
**PQE**	**B3**	**C2**		**C3**	**A3**
**Blastocyst-Stage**	**DET (double embryo transfer)**				**SET (single embryo transfer)**
	**GQB**		**PQB**		**/**
**GQB**	**E3**		**/**		**E1**
**FQB**	**E5**		**E4**		**E2**

GQE, good-quality embryo; AQE, average-quality embryo; PQE, poor-quality embryo; GQB, good-quality blastocyst; FQB, fair-quality blastocyst.

The evaluation of blastocysts was based on the classification of Gardner et al. (13): AA, AB, BA, and BB were defined as good-quality blastocyst (GQB), and BC and CB were defined as fair-quality blastocyst (FQB). Two embryologists double-checked all embryo evaluations.

The number of embryos transferred was determined by the physician’s approach, the patient’s desire, and other clinical factors such as clinical history and previous embryo transfer cycle outcomes.

### Outcomes

Live birth was defined as live birth of at least one baby at ≥28 weeks of gestation. LBR was calculated as the number of cycles with live birth/number of embryo transfer cycles. MBR was defined as the number of cycles with multiple births/number of clinical pregnancy cycles. Clinical pregnancy was defined as the presence of an intrauterine gestational sac and active fetal heartbeat.

### Statistical analysis

Baseline characteristics are presented as mean (standard deviation [SD]) for continuous variables and as percentage for categorical variables; these characteristics were compared between the groups using the chi-square test or analysis of variance (ANOVA). Bonferroni multiple comparison test was conducted to compare the groups between which statistically significant differences existed. Multivariate logistic regression analysis was performed to investigate the risk of different embryo combinations in multiple births. The results were reported as adjusted odds ratios (aORs) with 95% confidence intervals (CIs). All statistical analyses were performed using the two-sided 5% level of significance and the statistical package Stata version 12 (StataCorp, College Station, TX, United States).

## Results

We retrospectively collected the data on 10,060 FET cycles including 6,408 cycles of cleavage-stage embryo transfer and 3,652 cycles of blastocysts transfer between 2015 and 2020. The baseline characteristics of patients in all cohorts are summarized in **Table 2**. The most common indication for *in vitro* fertilization (IVF) was tubal factor (44.24%). The percentage of primary infertility was 54.9%, and the ovarian reserve indicator [anti-Müllerian hormone (AMH)] level ranged from 0.32 to 12.75 ng/ml which declined as age increased.

**Table 2:** Baseline characteristics of study cycles.

	<30 Years (N=3775)	31-35 Years (N=3744)	36-39 Years (N=1575)	>=40 Years (N=966)	P
BMI (kg/m2)					<.0001
<18.5	239 (6.3)	211 (5.6)	76 (4.8)	31 (3.2)	
18.5-23.9	2988(79.2)	2983 (79.7)	1290 (81.9)	803 (83.2)	
24-27	359 (9.5)	303 (8.1)	145 (9.2)	92 (9.5)	
>=27	189 (5.0)	247 (6.6)	64 (4.1)	40 (4.1)	
Type of infertility (%)					<.0001
Primary	2540 (67.3)	2104 (56.2)	641 (40.7)	237 (24.5)	
Secondary	1235 (32.7)	1640 (43.8)	934 (59.3)	729 (75.5)	
AMH	6.0±4.10	5.1±3.85	3.6±3.05	2.2±2.16	<.0001
Indication n (%)					<.0001
Tubal factor	1666 (44.1)	1670 (44.6)	736 (46.7)	376 (38.9)	
Endometriosis	226 (6.0)	202 (5.4)	108 (6.9)	61 (6.3)	
Ovulation disorders	109(2.9)	92 (2.5)	58 (3.7)	32 (3.3)	
Male factor	597 (15.8)	548 (14.6)	196 (12.4)	118 (12.2)	
Unexplained	223 (5.9)	199 (5.3)	100 (6.3)	66 (6.8)	
combination	954 (25.3)	1033 (27.6)	377 (23.9)	313 (32.4)	

Data are expressed as mean ± SD or as percentage of women (percentage) for categorical variables. †One-way ANOVA for continuous variables and chi-squared test for categorical variables.

BMI, body mass index; AMH, anti-Müllerian hormone.

### Age distributions of FET cycles

The group-wise distribution based on women’s age is presented in **Table 3**. For women aged ≥40 years, the proportion of single-cleavage embryo transfer cycles (A1, A2, and A3 groups) was 21.2%, while for those aged <30 years, the proportion was only 4.5%. Additionally, for all age groups, double AQE transfer cycles (C1 group) accounted for the highest proportion, followed by single poor-quality blastocyst (PQB) transfer cycles (E2 group).

**Table 3:** Distribution of groups based on the number and quality of embryos transferred in women of different ages.

	<30 Years(N=3,775)	31−35 Years(N=3,744)	36−39 Years(N=1,575)	>=40 Years(N=966)	P	Heat Map
	**n (%)**	**n (%)**	**n (%)**	**n (%)**		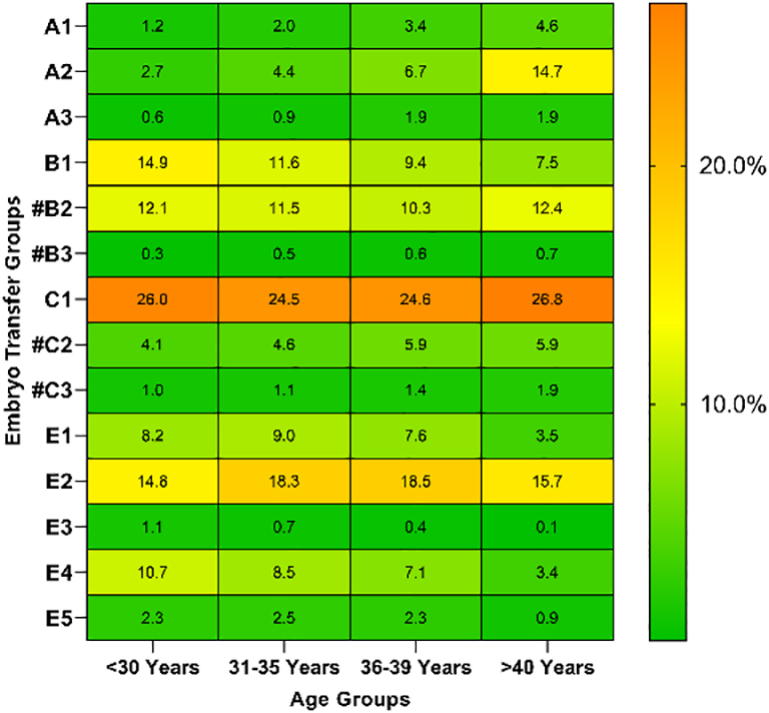
A1	46 (1.2)	74 (2.0)	53 (3.4)	44 (4.6)	<0.001	
A2	103 (2.7)	164 (4.4)	105 (6.7)	142 (14.7)	<0.001	
A3	22 (0.6)	32 (0.9)	30 (1.9)	18 (1.9)	<0.001	
B1	562 (14.9)	433 (11.6)	148 (9.4)	72 (7.5)	<0.001	
B2	457 (12.1)	430 (11.5)	162 (10.3)	120 (12.4)	0.2407	
B3	12 (0.3)	20 (0.5)	9 (0.6)	7 (0.7)	0.2829	
C1	983 (26.0)	918 (24.5)	388 (24.6)	259 (26.8)	0.2798	
C2	155 (4.1)	171 (4.6)	93 (5.9)	57 (5.9)	0.0102	
C3	36 (1.0)	43 (1.1)	22 (1.4)	18 (1.9)	0.1033	
E1	311 (8.2)	336 (9.0)	119 (7.6)	34 (3.5)	<0.001	
E2	557 (14.8)	687 (18.3)	292 (18.5)	152 (15.7)	<0.001	
E3	42 (1.1)	26 (0.7)	6 (0.4)	1 (0.1)	0.0016	
E4	404 (10.7)	318 (8.5)	112 (7.1)	33 (3.4)	<0.001	
E5	85 (2.3)	92 (2.5)	36 (2.3)	9 (0.9)	0.0376	

### LBR and MBR based on different embryo transfer groups

Comparison of LBRs and MBRs among the cycles is presented in **Table 4**. Significant differences were observed among LBRs or MBRs in all cleavage embryos and single blastocyst transfer cycles. However, no significant difference was observed in the double blastocyst transfer cycle.

**Table 4:** Live birth rate and multiple birth rate of different embryo transfer groups.

	Live birth rate n/N(%)	P	Multiple birth rate n/N(%)	P
A1	49/217 (22.6)	*X^2^=* 9.9179 P=0.0070	--	--
A2	83/514 (16.1)		--	
A3	9 /102 (8.8)		--	
B1	547/1215 (45.0)	*X^2^=* 105.0364 P<0.001	261 /643 (40.6)	*X^2^=* 35.7663 P <0.001
B2	438/1169 (37.5)		177 /527 (33.6)	
B3	14 /48 (29.2)		6 /19 (31.6)	
C1	860/2548 (33.8)		311/1038 (30.0)	
C2	104 /476 (21.8)		30 /136 (22.1)	
C3	24/119 (20.2)		3/30 (10.0)	
E1	389 /800 (48.6)	*X^2^=* 38.0476 P<0.001	--	
E2	602/1688 (35.7)		--	
E3	39/75 (52.0)	*X^2^=* 3.6147 P< 0.1641	19 /45 (42.2)	*X^2^=* 1.0215 P=0.6001
E4	446 /867 (51.4)		246/543 (45.3)	
E5	130 /222 (58.6)		73 /148 (49.3)	

### LBRs and MBRs based on different embryo transfer groups and ages

LBRs and MBRs based on embryo transfer groups and age are shown in **Tables 5**, **6**. Overall, for each embryo combination, LBRs and MBRs decreased with increasing maternal age. The highest LBR and MBR were 64.7% and 54.8%, respectively, both of which were noted in women aged <30 years receiving DET-GQB+PQB transfer. For those aged ≥40 years, the highest LBR was 35.3% in the SET-GQB group and 25% in the DET-GQE group.

**Table 5:** Live birth rate of different embryo transfer groups based on women’s age.

	<30 Years n/N(%)	31−35 Years n/N(%)	36−39 Years n/N(%)	>=40 Years n/N(%)	Heatmap
A1	15/46 (32.6)	23/74 (31.1)	10/53 (18.9)	1/44 (2.3)	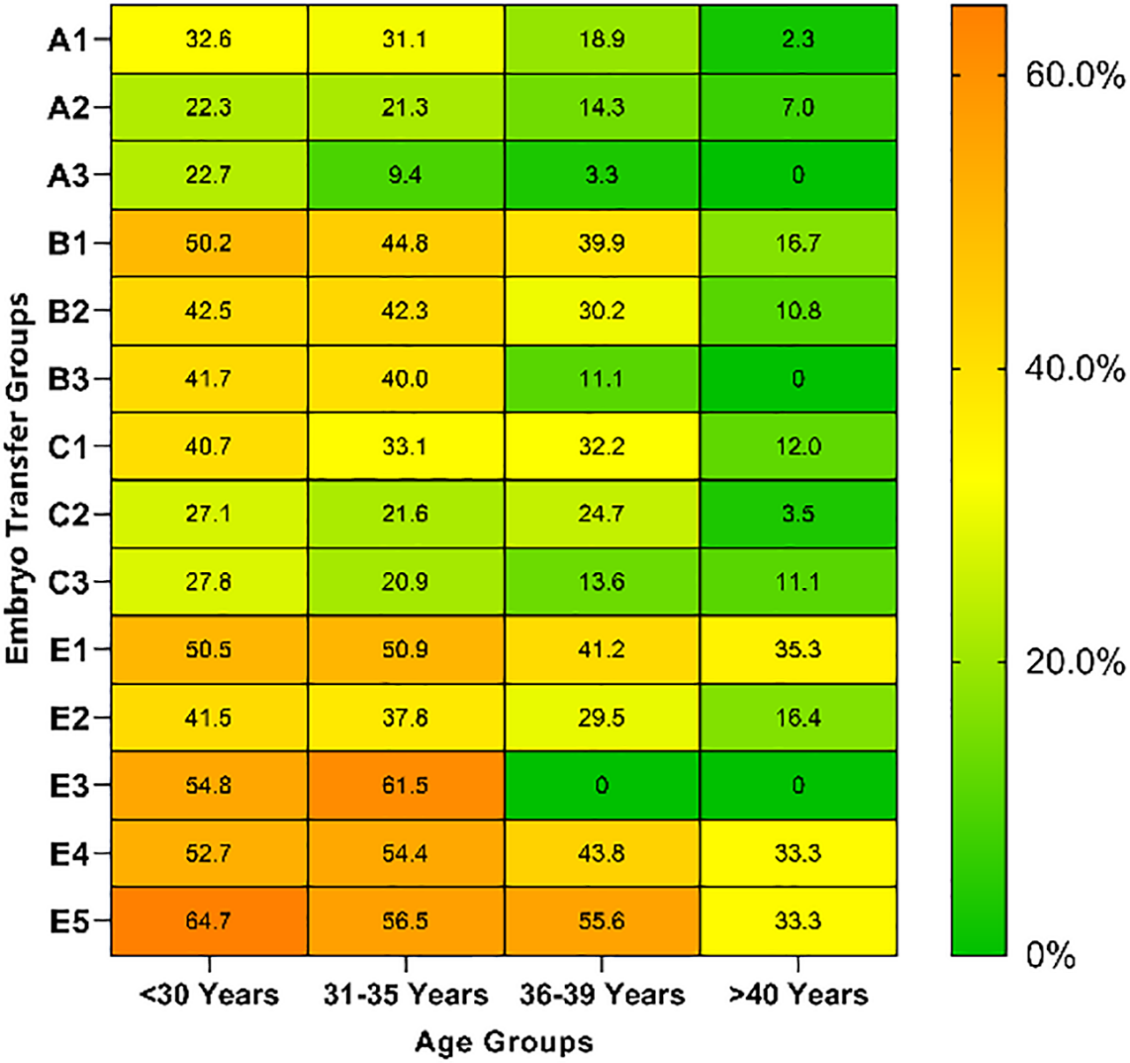
A2	23/103 (22.3)	35/164 (21.3)	15/105 (14.3)	10/142 (7.0)	
A3	5/22 (22.7)	3/32 (9.4)	1/30 (3.3)	0/18 (0.0)	
B1	282/562 (50.2)	194/433 (44.8)	59/148 (39.9)	12/72 (16.7)	
B2	194/457 (42.5)	182/430 (42.3)	49/162 (30.2)	13/120 (10.8)	
B3	5/12 (41.7)	8/20 (40.0)	1/9 (11.1)	0/7 (0.0)	
C1	400/983 (40.7)	304/918 (33.1)	125/388 (32.2)	31/259 (12.0)	
C2	42/155 (27.1)	37/171 (21.6)	23/93 (24.7)	2/57 (3.5)	
C3	10/36 (27.8)	9/43 (20.9)	3/22 (13.6)	2/18 (11.1)	
E1	157/311 (50.5)	171/336 (50.9)	49/119 (41.2)	12/34 (35.3)	
E2	231/557 (41.5)	260/687 (37.8)	86/292 (29.5)	25/152 (16.4)	
E3	23/42 (54.8)	16/26 (61.5)	0/6 (0.0)	0/1 (0.0)	
E4	213/404 (52.7)	173/318 (54.4)	49/112 (43.8)	11/33 (33.3)	
E5	55/85 (64.7)	52/92 (56.5)	20/36 (55.6)	3/9 (33.3)	

**Table 6:** Multiple birth rates of embryo transfer groups based on maternal age.

	<30 Years n/N(%)	31−35 Years n/N(%)	36−39 Years n/N(%)	>=40 Years n/N(%)	Heatmap
B1	142/239 (43.2)	89/223 (39.9)	25/71 (35.2)	5/20 (25.0)	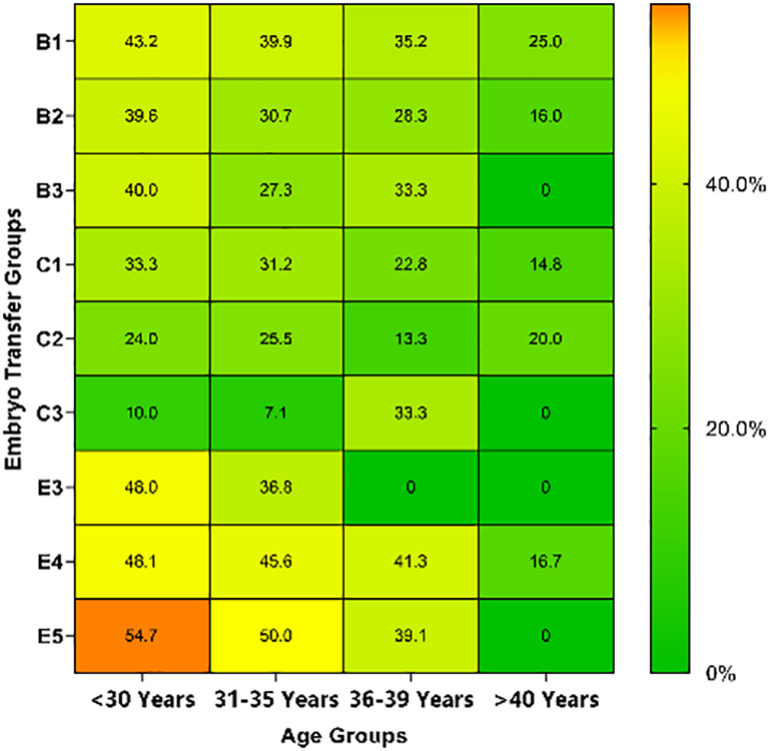
B2	91/230 (39.6)	65/212 (30.7)	17/60 (28.3)	4/25 (16.0)	
B3	2/5 (40.0)	3/11 (27.3)	1 /3 (33.3)	0/0 (0.0)	
C1	151/454 (33.3)	116/372 (31.2)	36/158 (22.8)	8/54 (14.8)	
C2	12/50 (24.0)	13/51 (25.5)	4/30 (13.3)	1/5 (20.0)	
C3	1/10 (10.0)	1/14 (7.1)	1/3 (33.3)	0/3 (0.0)	
E3	12/25 (48.0)	7/19 (36.8)	0/1 (0.0)	0/0 (0.0)	
E4	124/258 (48.1)	93/204 (45.6)	26/63 (41.3)	3 /18 (16.7)	
E5	35/64 (54.7)	29/58 (50.0)	9/23 (39.1)	0/3 (0.0)	

### Multiple comparisons of LBRs and MBRs based on embryo transfer groups and ages

Multiple comparisons with Bonferroni correction were performed, and groups involving a very small number of cases to be analyzed (B3, C3, and E3) were excluded. As shown in **Tables 7**, **8**, for women <30 years, the double AQE and AQE+PQE groups showed similar MBRs, whereas the double AQE group had a significantly higher LBR. In women aged 31−35 years, the double GQE and GQE+AQE groups had similar LBRs, with a higher MBR noted in the double GQE group. In women aged 36−-39 years, the double GQE group had a similar LBR but a higher MBR compared with the double AQE or AQE+PQE groups. In cycles of blastocyst transfer, GQB+PQB transfer (E5) had a similar LBR as SET-GQB transfer (E1), regardless of age. When we compared the SET-PQB (E2) and double PQB transfer (E4) groups, the DET group had a higher LBR in women aged <35 years but not in those aged >36 years. In women aged >40 years, no significant difference was noted in either MBRs or LBRs among either of the two groups, regardless of cleavage embryo or blastocyst transfer.

**Table 7:** Multiple comparisons of live birth rate in embryo transfer groups with p-value adjustments by Bonferroni correction.

Maternal age (years)	Cleavage embryo transfer (B1, B2, C1, and C2)				Blastocyst transfer (E1, E2, E4, and E5)			
**<30 Years**	**B1**	**B2**	**C1**	**C2**	**<30 Years**	**E2**	**E4**	**E5**
**A1**	0.214	1.000			**E1**	0.298	1.000	0.544
**A2**			0.001	1.000	**E2**		**0.016**	0.002
**B1**		0.140	0.003	<0.001	**E4**			1.000
**B2**			1.000	0.005				
**C1**				0.009				
**31−-35 Years**	**B1**	**B2**	**C1**	**C2**	**31–35 Years**	**E2**	**E4**	**E5**
**A1**	0.262	0.681			**E1**	**0.002**	1.000	1.000
**A2**			0.020	1.000	**E2**		**<0.001**	0.020
**B1**		1.000	0.000	<0.001	**E4**			1.000
**B2**			0.011	<0.001				
**C1**				0.022				
**36−39 Years**	**B1**	**B2**	**C1**	**C2**	**36–39 Years**	**E2**	**E4**	**E5**
**A1**	0.043	1.000			**E1**	0.656	1.000	1.000
**A2**			0.001	0.646	**E2**		0.204	0.071
**B1**		0.770	0.974	0.151	**E4**			1.000
**B2**			1.000	1.000				
**C1**				1.000				
**>=40 Years**	**B1**	**B2**	**C1**	**C2**	**>=40 Years**	**E2**	**E4**	**E5**
**A1**	0.099	0.690			**E1**	0.550	1.000	1.000
**A2**			1.000	1.000	**E2**		1.000	1.000
**B1**		1.000	1.000	0.134	**E4**			1.000
**B2**			1.000	0.985				
**C1**				0.431				

**Table 8:** Multiple comparison of multiple birth rate for embryo transfer groups based on maternal age.

Maternal age (years)	Embryo transfer groups			
**<30 Years**	**B2**	**C1**	**C2**	**E5**
**B1**	0.396	0.005	0.010	
**B2**		0.103	0.039	
**C1**			0.184	
**E4**				0.343
**31–35 Years**	**B2**	**C1**	**C2**	
**B1**	0.044	0.030	0.055	
**B2**		0.896	0.468	
**C1**			0.408	
**E4**				0.552
**36–39 Years**	**B2**	**C1**	**C2**	
**B1**	0.401	0.049	0.026	
**B2**		0.394	0.113	
**C1**			0.246	
**E4**				0.858
**>=40 Years**	**B2**	**C1**	**C2**	
**B1**	0.453	0.307	0.815	
**B2**		0.891	0.827	
**C1**			0.758	
**E4**				0.552

### Risk of multiple birth based on age and embryo transferred

**Figure 1:**
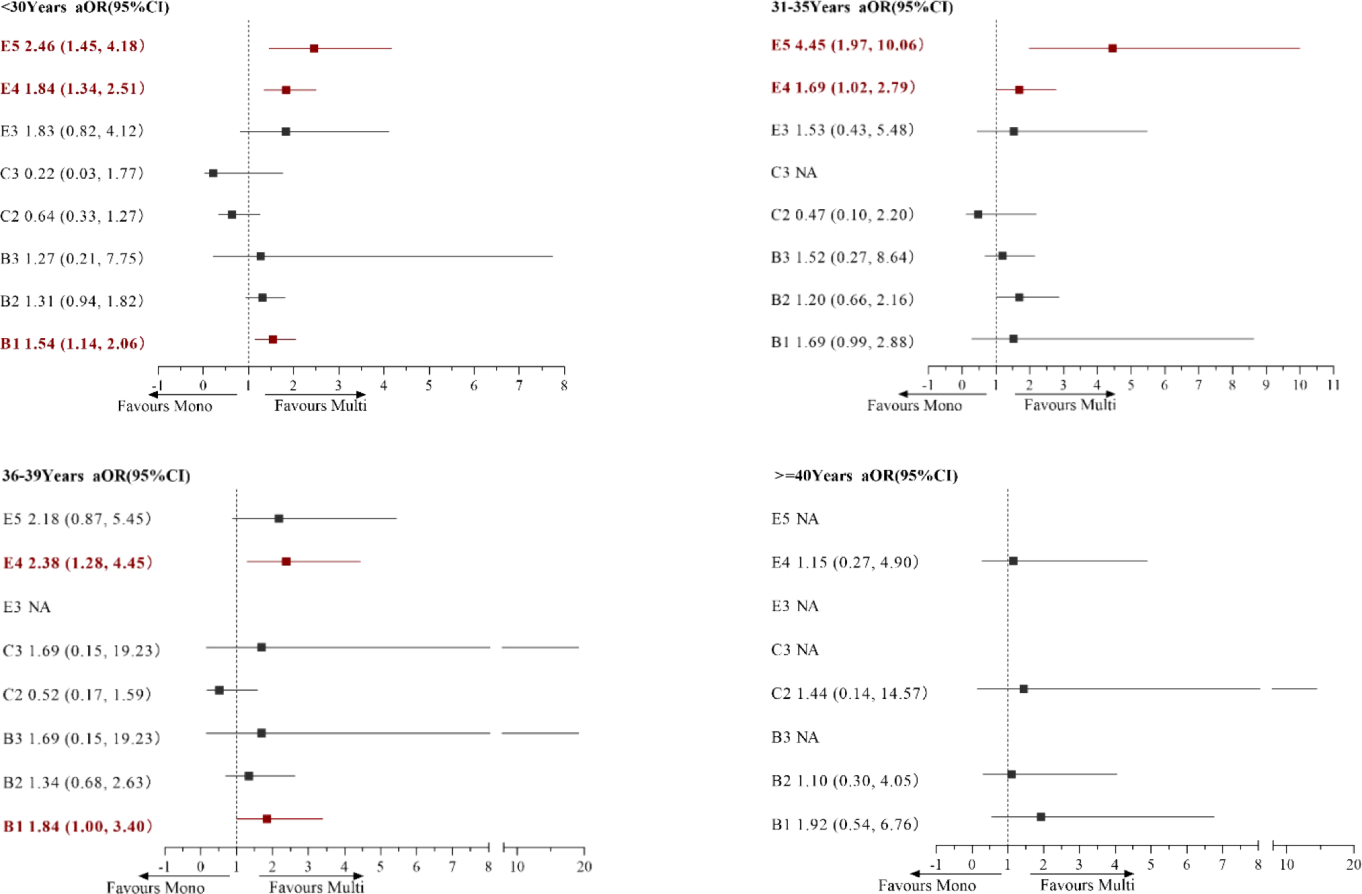
Logistic regression analysis for multiple birth rate (MBR) among patients based on age and embryo transfer group. *AOR: adjusted odds ratio for type of infertility, indications for IVF, body mass index (BMI), and anti-Müllerian hormone (AMH) in logistic regression analysis.

**Figure 1** presents the adjusted ORs for associations between the combinations of transferred embryos and multiple births in women of different ages. Multivariate logistic analysis revealed that the transfer of double GQE was an independent predictor for multiple birth (MB) in women aged <30 years and those aged 36–39 years [<30 years: aOR=1.54 (95% CI: 1.14-2.06, P < 0.01); 36−39 years: aOR=1.84 (95% CI: 1.0−3.4, P < 0.01)]. DET-PQB was positively associated with MB in women aged <40 years, and this effect remained statistically significant in the multivariate analysis (P < 0.01). In addition, for women aged <36 years, the transfer of GQB+PQB was an independent predictor for MB (<30 years: aOR=2.46 (95% CI: 1.45−4.18, P < 0.01); 31–35 years: aOR=4.45 (95% CI: 1.97−10.06, P < 0.01)].

## Discussion

### Principal findings

Our results analyzed the LBRs and MBRs in a variety of transferred embryo combinations in women of different ages. In women ≤ 35 years old, transfer of single good quality embryo, either cleavage or blastocyst, could result in an LBR comparable to double embryo transfer. In those older than 40 years, double embryo transfer could shorten the time interval to achieve live birth with relatively low MBR, except for good quality blastocyst available. However, for women between 36-40 years, the selection of transferred embryo should be dependent on the quality of all available embryos.

### Results in the context of what is known

In recent years, the goal of ART has been to achieve a healthy singleton gestation. The American Society for Reproductive Medicine guidelines recommends that patients aged ≤37 years should be encouraged to undergo SET regardless of the embryo stage (14). In 2018, an expert consensus formulated by the Committee of Chinese Society of Reproductive Medicine suggested that SET in the first embryo transfer cycle and ≤2 embryos should be transferred in each cycle, regardless of age (15). Despite this, the SET strategy is not widely accepted in China, and clinicians face the challenge of reducing MBR without impairing LBR in couples receiving ART treatments.

In advanced age of women, the proportion of single good cleavage embryo transfer cycles increased (from 1.2% in 30y to 4.6% in ≥40y), while the proportion of single GQB and two GQE decreased (from 8.2% and 14.9% in 30y to 3.5% and 9.4% in ≥40y, respectively). This was due to the low number of available embryos or intentional SET to avoid multiple pregnancies due to previous cesarean section or family planning. The distribution of embryo grades in the cleavage phase was comparable among women of different ages. However, the proportion of high-quality transferred blastocysts decreased with increasing maternal age. The reasons for the lower blastocyst formation rate in older women include increased aneuploidy rate, mitochondrial genome D-loop loci mutations, and low levels of stored maternally transcribed mRNA, which is involved in trophectoderm function and maintenance of the blastocoel (16). Blastocyst formation from good-morphology embryos has been reported to decrease significantly from 66.9% in women aged <35 years to 53% in those aged >39 years (17). As an embryo selection technique, blastocyst culture does not improve embryo viability, but can elevate the efficiency of embryo transfer, especially in advanced-age women.

A meta-analysis published in 2022, including 14 randomized controlled studies, focused on the association of LBR and MBR with the age or number of embryos transferred (18). Overall, the probability of live birth and multiple pregnancies decreased in women of advanced age or those receiving SET. However, only few studies (18, 19) have provided suggestions on how to make the best use of available embryos, based on both age and embryo grade. Here, we performed multiple comparisons and logistic regression analyses to explore individualized embryo transfer strategies. Give that the cases of GQE+PQE (B3), double PQE (C3), and double GQB (E3) were very few, these situations were considered as B2, C2, and E5, respectively, for convenience.

SET-GQB achieved an LBR similar to double blastocyst transfer (E3, E4, and E5) groups in women of all ages. Thus, SET is recommended when at least one GQB is available, which is consistent with the American Society for Reproductive Medicine guidelines (20). Some studies have suggested that compared with a single GQB transfer, DET-GQB+PQB results in almost the same LBR at the expense of a marked increase in the likelihood of multiple pregnancies (21, 22). However, Zhu et al. reported inconsistent findings, which showed a higher LBR after double GQB than after single GQB, with an adjusted OR of 1.76 (CI: 1.20, 2.57) (23). A policy of selective blastocyst culture or patient age may result in diversity. The more valuable meaning of SET is the sharply decreased MBR and improved CLBR in consecutive transfer cycles. It has been reported that two cycles of single GQB transfer could reach an LBR of 48.5% and a CLBR of 64.7%, whereas the LBR of double GQB transfer was only 48.9% (24).

Somewhat unexpectedly, the transfer of double PQB (E4) did not result in compromised LBR or MBR compared with the transfer of GQB + PQB (E5), indicating that the replacement of a good-quality blastocyst with a poor one in the double blastocyst transfer cycle did not compromise the chances of live birth. Based on a report enrolling 2,582 blastocyst transfer cycles from China, the transfer of double PQB and GQB+PQB had similar LBR and MBR, regardless of the age of ≤37 years or older (25). Theodorou et al. also reached the same conclusion (26). It seems that the predictive value of the morphology score for LBR and MBR was diminished when double blastocysts were transferred. Thus, the DET of blastocysts should be avoided, except for women older than 40 years, and when no GQBs are available.

In the cycles of cleavage embryo transfer, the relationship between embryo grade and LBR and MBR depended on the age of the women. For women aged <35 years, the addition of another embryo is not helpful for LBR when good-quality embryos are available. However, in women 36-40 years of age, double GQE transfer could increase the LBR compared with SET. Thus, DET-GQE should be avoided in patients aged ≤35 years and should be performed in those aged 36–40 years after full consultation. In addition, considering the CLBR and MBR risk, the combination of high- and low-quality embryos is preferred to DET-GQE in women aged ≤40 years, as the reserved high-grade embryo could bring more chances of LBR in the following embryo transfer cycle. In fact, for women with poor prognosis and decreased ovarian reserve, cumulative LBR was more important than LBR of a single cycle (27).

### Clinical implications

The proposed embryo transfer practices based on different ages are listed as follows.

**Table:** 

	Cleavage embryo	Blastocyst
≤35 years	SET-GQE>DET-AQE>DET-AQE+PQE>DET-PQE	SET-QGB>SET-PQB >DET-PQB
36**–**40 years	DET-GQE+AQE>DET-AQE>DET-AQE+PQE>SET-GQE	
>40 years	DET with embryos as high grade as possible	SET-QGB>DET-PQB> SET-PQB

The principle is that, in the case of similar LBRs, embryo combinations with lower MBRs are preferred, and in the case of similar MBRs, embryo combinations with higher LBRs are preferred.

For women aged >40 years, the relationship between transferred embryos and LBR and MBR becomes indistinct, which is mainly related to the high rate of embryo chromosomal abnormalities (28–30). Preimplantation genetic testing for aneuploidy (PGT-A) is the best choice for women of advanced age, but it requires specific qualifications in China. Blastocyst culture is another good strategy because the aneuploidy rate is much lower in high-quality blastocysts than in high-quality D3 embryos (31). The elimination of cleavage embryos with poor developmental potential through blastocyst culture may increase the efficacy of embryo transfer. Chen et al. (32) reported that blastocyst culture and transfer did not increase the CLBR in women aged ≥38 years, but significantly increased the pregnancy rate per embryo transfer cycle. In 2012, data from 32,732 cycles with double embryos transferred were analyzed, which showed that the ORs and absolute risk differences for multiple births, preterm births, and low birth weight were all smaller in women ≥40 years than in younger women (33). Thus, if only cleavage embryos are available, DET should be suggested in women older than 40 years, as it tends to achieve higher LBR per cycle and shorten the time to achieve a live birth.

### Research implications

The currently used criteria for embryo morphological score are of very limited value for women with advanced age. Thus, novel non-invasive methods for embryo development should be investigated to improve the efficiency of embryo transfer.

### Strengths and limitations

To the best of our knowledge, this is one of the largest retrospective studies evaluating LBR and MBR based on maternal age and embryo grade in both cleavage-stage embryo and blastocyst transfer cycles. However, our study was limited by its retrospective design, and the fact that it was performed at a single center. Another major limitation was the decision of the number of the transferred embryos which was influenced by complicated factors from both physicians and infertile couples. Moreover, we did not exclude patients with repeated implantation failure (RIF) associated with adverse pregnancy outcomes. In addition, other potential confounders such as the pre-thrombotic state and polycystic ovary syndrome (PCOS) were not analyzed.

## Conclusions

Based on the current evidence, SET should be selected for high-quality blastocysts in women of all ages. When good-quality cleavage embryos are available, the choice of SET or DET with GQE or AQE should depend on the age of the woman. For elderly women aged ≥40 years, if no GQB is available, DET with embryos as high a grade as possible is recommended. It should be noticed that since our data were from FET cycles, its validation in fresh cycles needs more evidence. Overall, the choice of embryo transferred should be jointly made by patients and physicians based on individualized transfer strategies, which need to be verified by further high-quality randomized controlled trials or national registry-based cohort studies.

## Data availability statement

The relevant data involves patient privacy, so it is not disclosed or uploaded to the database. Requests to access the datasets should be directed to Zhi-hong Niu.

## Ethics statement

The studies involving human participants were reviewed and approved by Ruijin Hospital Ethics Committee Shanghai JiaoTong University School of Medicine. The patients/participants provided their written informed consent to participate in this study.

## Author contributions

XW and W-JZ contributed equally to this work. Z-HN and A-JZ designed the study. B-FX, QC, LX,H-HX and Z-HN collected data and participated in parts of analysis of the data. XW and W-JZ analyzed data and wrote the paper. All authors contributed to the article and approved the submitted version.
